# Radiation Exposure in Patients with Isolated Limb Trauma: Acceptable or Are We Imaging Too Much?

**DOI:** 10.3390/jcm9113609

**Published:** 2020-11-09

**Authors:** James A. Wheeler, Natasha Weaver, Zsolt J. Balogh, Herwig Drobetz, Andrew Kovendy, Natalie Enninghorst

**Affiliations:** 1 Lismore Base Hospital, Lismore, New South Wales 2480, Australia; James.wheeler@health.qld.gov.au (J.A.W.); herwig.drobetz@newcastle.edu.au (H.D.); 2 School of Medicine and Public Health, University of Newcastle, Callaghan, New South Wales 2308, Australia; Natasha.Weaver@newcastle.edu.au (N.W.); natalie.enninghorst@newcastle.edu.au (N.E.); 3 John Hunter Hospital, Newcastle, New South Wales 2305, Australia; 4 North Coast Cancer Institute, North Coast, New South Wales 2450, Australia; Andrew.Kovendy@health.nsw.gov.au

**Keywords:** orthopaedic, trauma, radiation, safety, imaging, extremity, fracture

## Abstract

The aim of our study was to investigate the cumulative effective dose of radiation resulting from medical imaging in orthopaedic patients with isolated extremity trauma. Deidentified radiology records of consecutive patients without age restriction with isolated extremity trauma requiring operative treatment at a regional hospital were reviewed retrospectively over a 1-year period, and the effective dose per patient for each study type of plain film X-ray, computed tomography, and operative fluoroscopy was used to calculate cumulative effective dose. Values were summarised as mean, ± standard deviation, maximum, and proportion with overdose (>20 mSv). The study cohort included 428 patients (193 male and 235 female) with an average age of 44 years (±28). There were 447 procedures performed, i.e., all involved operative fluoroscopy, 116 involved computed tomography, and 397 involved X-ray. The mean cumulative effective dose per patient was 1.96 mSv (±4.98, 45.12). The mean cumulative effective dose for operative fluoroscopy was 0.32 mSv (±0.73, 5.91), for X-ray was 1.12 mSv (±3.6, 39.23) and for computed tomography was 2.22 mSv (±4.13, 20.14). The mean cumulative effective dose of 1.96 mSv falls below the recommended maximum annual exposure of 20 mSv. This study can serve as a guide for informing clinicians and patients of the acceptable radiation risk in the context of isolated extremity trauma.

## 1. Introduction

The information gained from medical imaging in the management of trauma patients is invaluable, however, there is evidence to support that excessive amounts of radiation may cause various types of cancers [1,2,3,4]. Imaging modalities produce different amounts of ionizing radiation. A knee radiograph has an average effective dose of 0.005 millisieverts (mSv) versus 0.05 mSv for a chest radiograph. A single computed tomography (CT) trauma scan, which involves imaging the head, neck, chest, abdomen and pelvis, can have an average effective dose of 34 mSv or the equivalent of 680 chest radiographs [5].

In Europe and Australia, the permissible annual exposure dose of radiation workers cannot exceed 20 mSv. This is determined by the International Commission of Radiological Protection (ICRP) [2,4]. Within the clinical setting, there are no statutory limits on the amount of radiation exposure to patients from medical imaging procedures.

The average background radiation exposure for any individual in the community is approximately 3 mSv per year [1,6]. Comparatively, a 7-h airplane trip exposes passengers to 0.02 mSv, climbing to the summit of Mount Everest is 1 mSv, or smoking 30 cigarettes per day for a year induces 36 mSv [5]. Exposure to 1 sievert (Sv) (1000 mSv) creates a relative risk of 1.6 for the development of solid cancer [7], meaning that a cumulative radiation exposure of 1 Sv can increase an individual’s risk of developing a solid cancer at any age by 60%. Furthermore, it has been estimated that the same level of radiation exposure will increases a person’s absolute risk of mortality from cancer later in life by 5% [4]. Therefore, if an individual is exposed to the ICRP radiation exposure limit of 20 mSv per year, it will take 50 years for that person to be exposed to 1 Sv of radiation and its associated 60% increased risk of developing any solid form of cancer and the additional 5% absolute risk of mortality from cancer [4]. 

The stochastic effects of radiation-induced cancer have a random probability distribution that may be analysed statistically, but not precisely predicted. As such tissue weighting factors are used to calculate the effective dose of radiation sensitivity of different organs. Although there is no dose threshold, these values are used to specify exposure limits to ensure the occurrence of stochastic health effects is kept below unacceptable levels (Table 1) [4]. Deterministic effects are characterised by a threshold dose and an increase in severity of reaction when the dose is increased. The calculation of dose imparted during diagnostic imaging are generally based on stochastic models [4,8,9].

Current literature reports that patients are being exposed to levels greater than 20 mSv. These studies include all trauma involving the axial skeleton, thereby including the most radiosensitive tissues [10,11,12]. There is limited research investigating radiation exposure of patients with orthopaedic extremity trauma. These patients receive multiple imaging procedures, however, the amount of absorbed radiation is less as the areas’ exposed are more radioinsensitive [13,14,15]. 

The primary aim of this study was to quantify the amount of radiation patients are exposed to who have isolated extremity trauma, from diagnosis to treatment. The authors hypothesised that radiation exposure of isolated limb trauma would be less than the recommended annual limit of 20 mSv as set out by the ICRP. 

## 2. Method

This is a retrospective cohort study, which included all patients of any age who presented to the Emergency Department (ED) with an isolated extremity injury at a regional Australian hospital. Patients’ were included if they had any form of diagnostic imaging performed in ED and operative fixation performed with fluoroscopy at the principle hospital. Extremity trauma was defined as any fracture from the proximal femur or clavicle proximally to the phalanx of the finger or toe distally. Exclusion criteria involved fractures of the pelvis or spine, diagnostic imaging performed outside the ED, operative fixation performed at another hospital, and multiple extremity injuries surgically repaired under the same general anaesthetic. Furthermore, CT trauma scans were excluded from analysis, as they irradiated the entire body and were not specific to the isolated limb trauma. Multiply injured patients who had surgical fixation under different general anaesthetics were included, as these separate procedures could be measured. The sample included all imaging for each patient, including CT, plain film X-ray (XR), and operative fluoroscopy (OF). 

A list of medical record numbers (MRN) was submitted to the Picture Archiving and Communication System (PACS) administrator of the main computer database. The specific radiation dose for each patient was generated automatically in units of mGy.cm^2^ for CT and Gy.cm^2^ for XR and OF. This represents total exposure based on imaging modality combined with specific patient demographics such as height, weight and sex, which is stored in the PACS database after every procedure. Through consultation with the radiology department within the Northern New South Wales Local Health District (NNSWLHD), the radiation dose values specific to the machine used was confirmed as being of the lowest possible patient exposure dose. These values where submitted to the Radiation Physicist of the NNSWLHD for conversion into mSv, using tables outlined in the ICRP 103 publication, which use tissue weighting factors to determine the sensitivity of various tissues to cancer induction (Table 1) [4]. The data were deidentified, and any identifiable data were not recorded. 

The study cohort included 428 patients (193 male and 235 female) with an average age of 44 years (±28, range (R) 3–99 years). There were 108 (25%) patients aged 17 years or younger. There were 447 procedures performed, 59% were injuries to the upper extremity and 41% were injuries to the lower extremity. All procedures involved OF, mean 41.5 s (±180.0; R 0.0–3660.0), 116 procedures involved at least one CT (±0.3; R 1–3) and 397 procedures involved at least one XR (3.9, ±2.2, R 1–25).

### 2.1. Ethics

This study was exempt from ethical review in accordance with Section 5.1.22 of the National Statement on Ethical Conduct in Human Research by being a negligible risk activity proposing to analyse already collected, deidentified data.

### 2.2. Statistics

Data were cleaned before analysis. Seven patients with polytrauma were excluded due to not being able to determine dose per injury. Total dose per patient and dose per procedure were dichotomised in order to calculate the proportion of overdoses (dose over the annual limit of 20 mSv). Quantitative variables were summarised as mean (M), standard deviation (±SD) and maximum. Qualitative variables were summarised as frequency count and percentage. Data were analysed in SAS 9.4 (SAS Institute, Cary, NC, USA)™ software.

## 3. Results

The mean CED per patient was 1.96 mSv (±4.98, 45.12), where CED was calculated as the total dose from OF, XR and CT for each patient. The distribution of this CED dataset is positively skewed (Table 2).

**Figure 1:**
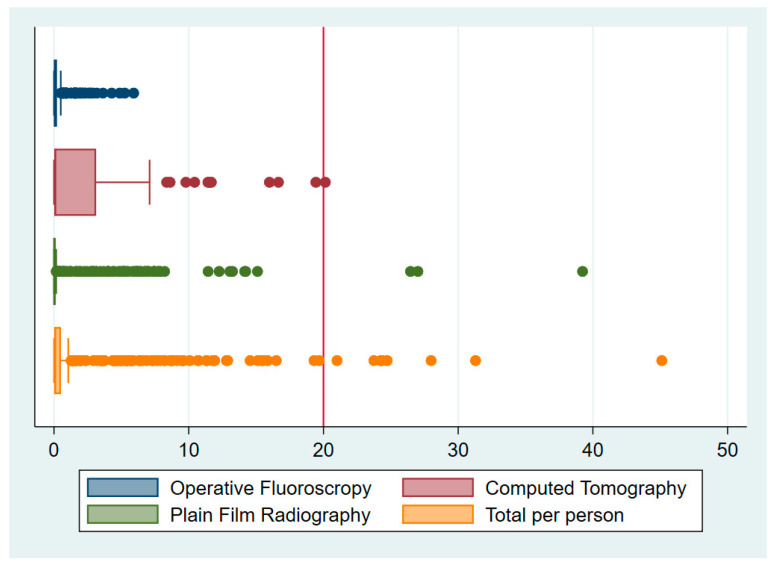
Radiation exposure by type.

There were 8 patients who had 10 procedures between them (less than 1.9% of the sample) that received in excess of 20 mSv (Table 2). Of these, 3 procedures received above 20 mSv from CT alone, 3 from XR, and 4 from a combination of all modalities (Table 3 and Figure 1). 

**Figure 2:**
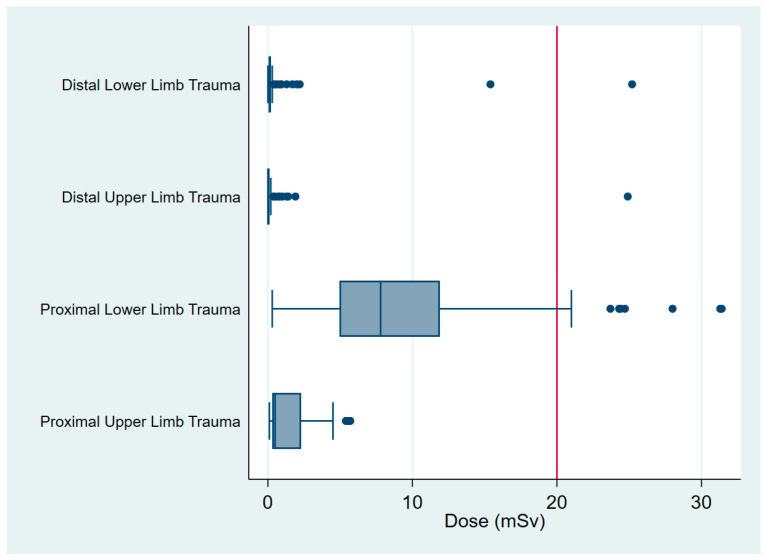
Radiation exposure by location.

**Figure 3:**
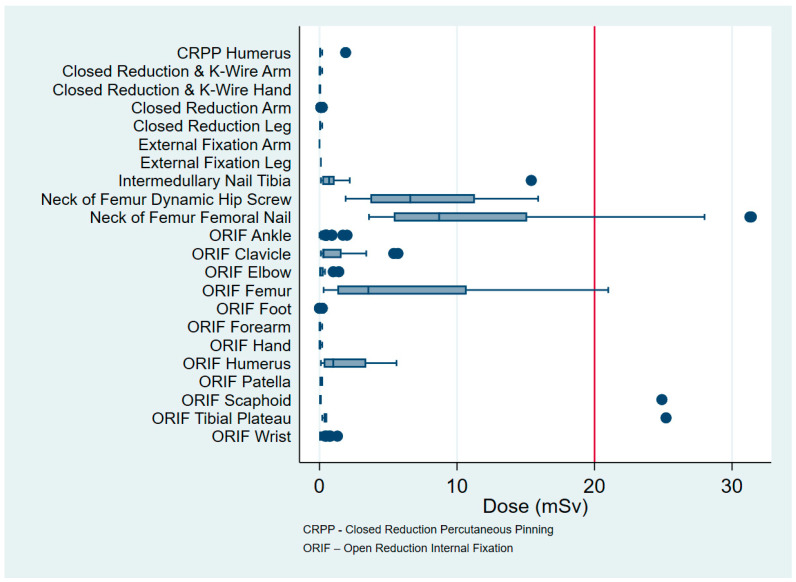
Radiation exposure by procedure.

The procedures involved 1 wrist scaphoid open reduction internal fixation (ORIF), 1 tibial plateau ORIF and 8 operations involving the proximal femur. There were no procedures of the proximal humerus or clavicle that received in excess than 20 mSv (Figure 2), and OF in isolation did not expose any patient to greater than 20 mSv (0.32, ±0.73, 5.91) (Figure 1). The mean radiation dose from XR was 1.12 mSv (±3.6, 39.23) and from CT was 2.22 mSv (±4.13, 20.14) (Table 2 and Figure 1). The maximum dose for each of these modalities involved imaging around the pelvis for fractures of the proximal femur (Table 4 and Figure 3). 

The three procedures that have the highest CED involve the proximal femur. Specifically, these are intermedullary nailing (11.57, ±7.78, 31.37), dynamic hip screw (7.7, ±4.03, 15.85) and ORIF (6.28, ±7.01, 21.01). The procedure with the lowest CED is closed reduction and casting of the arm (0.03, ±0.04, 0.24). Other notable procedures in order from cephalad to caudad are ORIF clavicle (1.3, ±1.93, 5.69), ORIF proximal humerus (1.77, ±1.81, 5.64), ORIF distal radius (0.11, ±0.21, 1.26), ORIF tibial plateau (3.48, ± 8.76, 25.15), intermedullary nailing of the tibia (1.91, ±4.29, 15.41), and ORIF of the ankle (0.22, ±0.36, 1.96) (Table 4 and Figure 3).

Of the 108 patients aged under 17 years, the mean CED was 0.14 mSv (±0.59, 5.69), the most common procedure being closed reduction distal radius (69 cases). Six patients received a CT (0.92, ±2.14, 5.28), with the youngest patient being aged 14 years (Table 4 and Figure 3).

## 4. Discussion

The findings from this study indicate that orthopaedic patients who suffer isolated limb trauma are exposed to levels of ionizing radiation, which pose an acceptable risk during their hospital admission. Most radiation exposure occurs in ED, during the preoperative phase of the patient’s presentation. Patients who sustain trauma of the proximal lower limb receive the highest CED. 

Kim et al. (2004) retrospectively reviewed the records of 46 patients during their intensive care unit admission and found that the mean CED was 106 mSv per patient, with a range of 11–289 mSv [11]. Prasarn et al. (2012) echoed these findings and investigated the CED of 1357 orthopaedic trauma patients during their inpatient hospitalization. Patients’ with Injury Severity Scores (ISS) greater than 16 received a mean of 48 mSv compared to those with an ISS below 16, receiving a mean of 23 mSv. Patients with spinal injuries received 15 mSv more than the patients with nonspinal injury [12]. Similar CED for spinal trauma was described by Martin et al. (2013), who investigated the CED of 406 patients with spinal fractures, of whom 59 had a spinal cord injury (SCI). Patients’ with SCI had a mean CED of 45 mSv compared to 38 mSv in those with spinal fractures [10]. 

These high values can be explained by the exposure of solid organs that are the most radiosensitive tissue to radiation. This is a strength of our study in that it focusses on extremity trauma. Additionally, individual patient CED’s were converted to specific values using tissue weighting factors outlined in the ICRP 103 publication, which accurately determines tissue sensitivity to cancer induction [4]. This has not been done in previous studies, which have used best estimations and raw CED values. 

A limitation of our study was the lack of ability to follow-up the patients post-procedure. This was because the imaging technology in the outpatient department was not the latest design and was unable to record patient-specific data. Further study limitations are its retrospective design, creating potential for inaccurate data entry, and patient diagnoses cannot be verified. Furthermore, the retrospective design may lead to case selection bias, in which a lower number of certain cases are analysed. Children often receive medical imaging at lower doses, and this may influence the overall mSv result of the cohort. OF dose is estimated based on optimal intraoperative imaging exposure. This is a common limitation to all studies, as it is nearly impossible to accurately measure OF dose. This is due to variables, such as distance of C-arm from patient, age of machine, operator variability, shape of operating room and the amount of personnel and airflow past the machine [16]. The data collected during this study were over a 12-month period. This resulted in two patients receiving more than one procedure due to separate traumatic incidences. As a result, these two patients received an annual CED in their dataset, which could be interpreted as abnormally high for that procedure in isolation. The conversion of dose area product into mSv may be controversial, as different doses produced by imaging modalities are converted using predetermined equations. This may lead to inaccuracies in results between facilities and should be acknowledged. 

The findings from our study demonstrate how influential radioinsensitive tissue is on the CED of isolated extremity trauma. Our data show that the maximum recommended dose is not reached during inpatient hospitalization and that common orthopaedic procedures involving ORIF of the distal radius and ankle have some of the lowest CED. Furthermore, the CED of clavicle and proximal humerus ORIF are low, given their proximity to central radiosensitive organs. Of note, fractures of the proximal femur have the highest CED, and this is largely due to radiation scatter and shoot through imaging around the pelvic organs and increased body habitus, to gain optimal imaging of the proximal femur. 

Clinicians need to be aware that radiation exposure is cumulative over time and that certain procedures have a higher CED than others. Future research could be conducted prospectively and include patient follow-up in the outpatient setting, to get a true representation of the amount of radiation exposure from diagnosis to complete recovery. 

## 5. Conclusions

The mean CED resulting from radiographic studies in our patient cohort was 1.96 mSv, which falls below the recommended annual exposure of 20 mSv. Procedures involving the proximal femur are associated with high CED, whereas common orthopaedic procedures such as distal radius and ankle ORIF have very low CED. The findings from this study demonstrate that multiple imaging procedures in the context of extremity trauma poses an acceptable radiation risk to patients. This study can serve as a guide for informing clinicians, patients and relatives emphasizing that the data are valid only to isolated limb trauma and not applicable to axial skeletal trauma or polytrauma patients.

## Figures and Tables

**Table 1:** K-values used for dose calculation (CT) mSv = K × mGy.cm^2^ and (XR and OF) mSv = K × Gy.cm^2^.

CT Dose		XR and OF Dose	
Site	K-Values Used	Site	K-Values Used
Pelvis	0.014	Ankle & Tibia/Fibula	0.01
Ankle	0.0002	Clavicle	0.036
Femur	0.0106	Elbow & Distal Humerus	0.01
Shoulder	0.00652	Femur	0.036
Elbow	0.00048	Fingers	0.01
Wrist & Hand	0.00022	Foot & Ankle	0.01
Hip	0.00730	Forearm & Elbow	0.01
Knee	0.00044	Hand & Wrist	0.01
Foot	0.00023	Hip & Proximal Femur	0.175
		Humerus	0.01
		Knee	0.01

**Table 2:** Total dose per patient by procedure.

Radiation Dose (mSv)	*n*	Mean	Std Dev	Maximum	Overdose (Number and Proportion above Annual Limit)
Operative fluoroscopy	428	0.32	0.73	5.91	0 (0.0%)
Computed tomography	116	2.22	4.13	20.14	1 (0.2%)
Plain film X-ray	397	1.12	3.60	39.23	3 (0.7%)
Cumulative effective dose	428	1.96	4.98	45.12	8 (1.87%)

**Table 3:** Procedures with dose over the annual threshold.

	Plain Film X-ray		Computed Tomography		Cumulative Effective Dose (XR + CT + OF)	
	*n*	Overdose (>20 mSv)	*n*	Overdose (>20 mSv)	*n*	Overdose (>20 mSv)
**Proximal upper limb**	30	0	27	0	30	0
**Distal upper limb**	235	0	234	1	235	1
**Proximal lower limb**	76	3	76	1	76	8
**Proximal lower limb**	106	0	105	1	106	1

**Table 4:** Dose by procedure.

	Operative Fluoroscopy					Computed Tomography				Plain Film X-ray				Total (OF + CT + XR)			
Procedure	N Obs	N	Mean	Std Dev	Max	N	Mean	Std Dev	Max	N	Mean	Std Dev	Max	N	Mean	Std Dev	Max
CR & K-wire arm	9	9	0.05	0.05	0.17	1	0.03	-	0.03	8	0.01	0.00	0.01	9	0.05	0.05	0.18
CR & K-wire hand	8	8	0.03	0.03	0.11	3	0.04	0.01	0.05	6	0.00	0.00	0.01	8	0.05	0.04	0.12
CR Distal Radius	79	79	0.03	0.04	0.19	0	-	-	-	79	0.01	0.01	0.06	79	0.03	0.04	0.24
CR Lower Limb	14	14	0.04	0.04	0.15	0	-	-	-	14	0.01	0.01	0.06	14	0.06	0.04	0.17
Ex-Fix Upper Limb	1	1	0.01	-	0.01	0	-	-	-	1	0.01	-	0.01	1	0.02	-	0.02
Ex-Fix Lower Limb	3	3	0.03	0.02	0.04	2	0.04	0.01	0.05	3	0.04	0.03	0.08	3	0.10	0.04	0.15
ORIF Femur	14	14	0.53	0.57	2.24	9	7.59	7.03	20.14	11	1.11	1.52	5.39	14	6.28	7.01	21.01
CRPP Humerus	16	16	0.17	0.46	1.90	0	-	-	-	16	0.01	0.00	0.01	16	0.18	0.46	1.90
ORIF Forearm	9	9	0.05	0.07	0.22	0	-	-	-	9	0.01	0.01	0.02	9	0.06	0.06	0.22
ORIF Humerus	15	15	0.25	0.39	1.55	9	1.98	1.57	4.20	13	0.38	1.00	3.69	15	1.77	1.81	5.64
ORIF Distal Radius	58	58	0.07	0.14	0.65	17	0.03	0.01	0.06	53	0.03	0.17	1.24	58	0.11	0.21	1.26
ORIF Hand	33	33	0.04	0.06	0.23	10	0.03	0.01	0.06	27	0.01	0.00	0.02	33	0.06	0.07	0.24
ORIF Elbow	12	12	0.13	0.27	0.97	3	0.62	0.69	1.39	12	0.01	0.00	0.01	12	0.29	0.46	1.42
ORIF Clavicle	15	15	0.13	0.15	0.58	6	2.53	2.41	5.28	15	0.16	0.12	0.43	15	1.30	1.93	5.69
ORIF Scaphoid	10	10	0.03	0.02	0.07	8	3.13	8.77	24.83	7	0.01	0.02	0.05	10	2.54	7.86	24.92
NOF DHS	19	19	0.86	0.76	2.54	6	3.38	1.84	7.09	18	6.10	3.77	14.15	19	7.70	4.03	15.85
Proximal Femur IMN	43	43	1.68	1.44	5.91	16	8.15	4.82	19.43	40	7.37	6.33	27.01	43	11.57	7.78	31.37
Tibia IMN	12	12	0.58	0.57	1.96	4	0.07	0.03	0.11	11	1.43	4.54	15.11	12	1.91	4.29	15.41
ORIF Tibial Plateau	8	8	0.25	0.15	0.48	6	4.24	10.09	24.83	6	0.07	0.11	0.30	8	3.48	8.76	25.15
ORIF Patella	3	3	0.06	0.09	0.17	0	-	-	-	3	0.07	0.11	0.20	3	0.14	0.10	0.21
ORIF Ankle	57	57	0.17	0.35	1.95	14	0.09	0.05	0.23	54	0.03	0.02	0.08	57	0.22	0.36	1.96
ORIF Foot	9	9	0.06	0.06	0.18	6	0.03	0.02	0.05	9	0.02	0.02	0.05	9	0.10	0.06	0.22

CR—Closed Reduction; Ex-Fix—External Fixation; ORIF—Open Reduction Internal Fixation; CRPP—Closed Reduction Percutaneous Pinning; NOF—Neck of Femur; DHS—Dynamic Hip Screw; IMN—Intramedullary Nail.
